# Functional biogeography of parasite traits: hypotheses and evidence

**DOI:** 10.1098/rstb.2020.0365

**Published:** 2021-11-08

**Authors:** Robert Poulin

**Affiliations:** Department of Zoology, University of Otago, PO Box 56, Dunedin 9054, New Zealand

**Keywords:** Bergmann's rule, body size, geographical range, niche breadth, latitude, Rapoport's rule

## Abstract

Functional biogeography, or the study of trait-based distributional patterns, not only complements our understanding of spatial patterns in biodiversity, but also sheds light on the underlying processes generating them. In parallel with the well-studied latitudinal diversity gradient, decades-old ecogeographical rules also postulate latitudinal variation in species traits. Notably, species in the tropics are predicted to have smaller body sizes (Bergmann's rule), narrower niches (MacArthur's rule) and smaller geographical ranges (Rapoport's rule) than their counterparts at higher latitudes. Although originally proposed for free-living organisms, these rules have been extended to parasitic organisms as well. In this review, I discuss the mechanistic hypotheses most likely to explain latitudinal gradients in parasite traits, and assess the empirical evidence obtained from comparative studies testing the above three rules as well as latitudinal gradients in other parasite traits. Overall, there is only weak empirical support for latitudinal gradients in any parasite trait, with little consistency among comparative analyses. The most parsimonious explanation for the existence of geographical patterns in parasite traits is that they are primarily host-driven, i.e. ecological traits of parasites track those of their hosts, with a direct influence of bioclimatic factors playing a secondary role. Thus, geographical patterns in parasite traits probably emerge as epiphenomena of parallel patterns in their hosts.

This article is part of the theme issue ‘Infectious disease macroecology: parasite diversity and dynamics across the globe’.

## 1. Introduction

Biogeography has revealed large-scale patterns in the distribution of species across space, and the underlying mechanisms and processes generating those patterns [1]. The best-studied and most ubiquitous biogeographic pattern across all living taxa has to be the latitudinal diversity gradient, characterized by an increase in species richness from the poles to the tropics [2–4]. Latitude serves as a convenient proxy for a range of bioclimatic factors, such as solar radiation and environmental stability, which together may act to favour and maintain high species diversity at low latitudes. Because of the universal influence of these factors, they should affect all organisms, including parasitic ones. Indeed, several biogeographic patterns first identified for free-living organisms have been found to apply also to parasites [5–7]. For instance, the latitudinal diversity gradient applies to at least some parasite taxa when species richness is measured *per host species* [8–10]. When parasite species richness is measured *per area* instead, the latitudinal diversity gradient holds well [11]. This is an almost inevitable consequence of the strong and universal positive relationship between host diversity and parasite diversity: areas with more diverse resources support a greater diversity of consumers, with congruent diversity across trophic levels being driven by ‘bottom-up’ processes [12]. This host–parasite link illustrates well how several aspects of parasite biogeography are likely dependent on host biogeography [13].

In recent years, focus has begun to shift from species-based to trait-based distributional patterns, or functional biogeography [14]. Here, I specifically refer to functional traits, which can be defined as any morphological, physiological, or ecological feature which indirectly impacts an organism's fitness and/or a species' long-term success [15]. Geographical variation in trait distribution within a clade provides insights into both past mechanisms of diversification and future responses of communities to environmental change [14,16]. For example, using functional trait diversity instead of taxonomic diversity allows a more discerning test of hypotheses proposed to explain the latitudinal diversity gradient [17]. It is therefore timely to revisit some old ecogeographical ‘rules’, proposed many decades ago, that claim to describe latitudinal gradients in species traits. For instance, Allen's rule states that among endotherms, the length of body extremities such as limbs and ears decreases toward higher latitudes [18,19], while Gloger's rule states that animals should have darker body coloration in warm and humid tropical areas than at higher latitudes [20].

Three ecogeographical rules regarding the latitudinal distribution of species traits have received particular attention. Firstly, Bergmann's rule proposes a latitudinal gradient in body sizes, such that among related species, those at higher latitudes generally achieve larger sizes on average [21]. Originally restricted to homeotherms, the pattern is thought to result from selection favouring larger sizes in colder environments, which minimizes heat dissipation through body surfaces by lowering surface-area-to-volume ratios [22]. However, it has been shown to apply widely to ectotherms, too, but through different mechanisms [23]. Secondly, another proposed latitudinal gradient applies to niche breadth or resource specialization, and is characterized by narrower species niches toward lower latitudes [24]. Since it originates from the writings of Robert MacArthur [25], it is hereafter referred to as MacArthur's rule. The rationale underlying this latitudinal gradient is that populations of resource species are more stable and temporally predictable in tropical areas, because of lower environmental variability and higher productivity, allowing consumers to specialize on fewer resources [24,26]. Thirdly, Rapoport's rule describes the positive correlation between latitude and the size of the geographical range of species [27]. More specifically, it is the latitudinal extent, i.e. the length of the north-south axis, of species' geographical ranges that increases positively as a function of the mid-point latitude of their range; the latitudinal gradient regarding the total surface area of geographical ranges is generally less clear [28]. The evidence for Rapoport's rule is considered equivocal, with the pattern being detectable mainly in the Northern Hemisphere [29].

**Figure 1.**
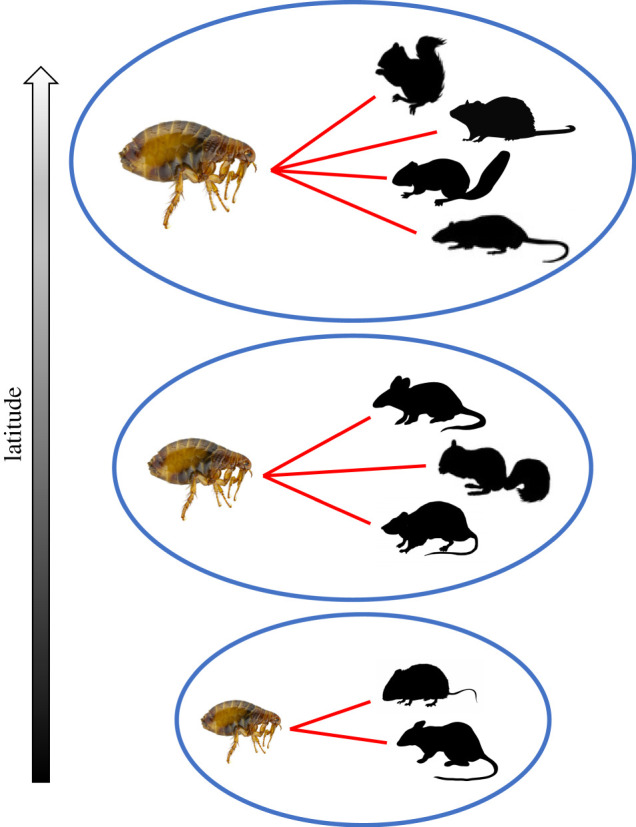
Schematic of the latitudinal gradients in parasite traits predicted by Bergmann's rule (body size), MacArthur's rule (niche breadth, or number of host species used) and Rapoport's rule (geographical range size; blue outline). (Online version in colour.)

Applied to parasitic organisms, these three ecogeographical rules (Bergmann's, MacArthur's and Rapoport's) pertain to three of the most important traits for parasite fitness and/or disease epidemiology: body size, host specificity (the number of host species that can possibly be used at a given life stage, a measure of parasite niche breadth), and parasite geographical range size, respectively. The first of these traits, parasite body size, is positively correlated with parasite fecundity across diverse parasitic taxa [30,31], whereas the other two properties play major and complementary roles in determining transmission success and population growth, as hedges against local extinction risk, and as determinants of zoonotic potential [32–34]. The three rules predict that, all else being equal, parasites should tend to be smaller-bodied, more host-specific, and have more restricted geographical ranges in the tropics than at higher latitudes (figure 1). Although latitudinal gradients in these three traits should apply equally to free-living and parasitic organisms, this may not necessarily be the case because of fundamental biological differences between hosts and parasites. However, the generality of ecogeographical rules among parasite taxa remains to be assessed.

In this short review, I evaluate the broad relevance of Bergmann's rule, MacArthur's rule and Rapoport's rule to metazoan parasites of animals. Firstly, I assess the empirical evidence for the three rules obtained from published comparative studies. Secondly, I discuss the mechanistic hypotheses proposed to explain the patterns observed, and identify those most likely to apply to parasites. Thirdly, I briefly summarize existing evidence for latitudinal gradients in other parasite functional traits. I then propose a robust framework for future tests of ecogeographical rules in parasites. Finally, I synthesize the findings into an integrated latitudinal gradient of parasite traits, to reveal what potential selective forces have shaped their differential evolutionary paths across the globe.

## 2. Parasite body sizes and Bergmann's rule

The original mechanism proposed to explain Bergmann's rule, i.e. that selection for large sizes at high latitudes served to reduce the surface-area-to-volume ratio and decrease heat loss [21,22], does not apply to ectothermic parasites. However, five alternative hypotheses or mechanisms, acting either alone or in combination, can potentially generate a latitudinal gradient in parasite body size matching that predicted by Bergmann's rule (table 1). One follows from the host's body size itself, whereas the others involve the direct action of environmental factors on the parasite.

**Table 1.** Main hypotheses and associated mechanisms explaining latitudinal gradients in functional traits of parasites.

hypothesis	how it works
*body size* (*Bergmann's rule*)	
tracking the host body size	parasite body size correlates positively with host body size; if host taxa follow Bergmann's rule, then so should their parasites
temperature-driven life rates	high temperature drives high metabolic rates, accelerates ageing and decreases lifespan, resulting in smaller achieved body sizes in the tropics
temperature-dependent cell sizes	somatic cells achieve larger sizes at lower temperatures, leading to larger body sizes in cold environments
latitudinal cline in parasite crowding	greater numbers of conspecific parasites per individual host in the tropics lead to increased competition, which selects for smaller body sizes
latitudinal cline in predation on parasites	more frequent and intense predation by cleaning organisms on an animal's ectoparasites in the tropics selects for predation avoidance and smaller body sizes
*host specificity* (*MacArthur's rule*)	
latitudinal cline in stability of host populations	the greater environmental stability and productivity of tropical ecosystems lead to more temporally stable and predictable host populations on which parasites can specialize
reduction of niche overlap	higher parasite diversity in the tropics leads to greater interspecific competition, and selects for a reduction in niche overlap through specialization on fewer host species
*geographical range* (*Rapoport's rule*)	
tracking the host(s) geographical range	the maximum range size of parasites corresponds to the overall geographical range of all their hosts combined, so that if hosts follow Rapoport's rule, then so would their parasites
latitudinal cline in climate variability	species in abiotically variable habitats at high latitudes have evolved wider environmental tolerances and expanded their geographical ranges further than species from relatively stable habitats in the tropics
*latitudinal gradient in egg size*	
body-size-dependent egg sizes	if parasite body sizes increase toward high latitudes (Bergmann's rule), so may their egg sizes
reducing temperature-dependent egg mortality	larger offspring at hatching have shorter development times and lower mortality risk, and should be favoured in colder and harsher climates

The size of the host places an upper physical limit on the body size that a parasite can achieve while allowing the host to survive and obtain food. Several comparative analyses have indeed demonstrated a positive interspecific relationship between parasite body size and host body size [31,35–37]. This covariation between parasite body size and host body size has also been elevated to the status of a rule, i.e. Harrison's rule [38]. As a consequence of this covariation, if host taxa follow Bergmann's rule, then, all else being equal, so should their parasites. The interesting question is whether parasites show a latitudinal gradient in body sizes independent of that of their hosts' sizes, i.e. whether other latitude-related effects act additively or synergistically with those of host size.

Indeed, other abiotic and biotic factors may also constrain parasite body sizes. Ambient temperature can drive metabolic and developmental rates, with higher temperatures causing organisms to age faster and live shorter lives, which may limit the size they can achieve [23]. For parasites, higher temperatures generally increase metabolic and reproductive rates [39], and may also select for smaller sizes. Alternatively, temperature can affect the size of an animal's individual cells, such that its overall body size is affected [40]. Ultimately, such temperature-driven mechanisms can only affect ectoparasites or parasites of ectotherms, since endoparasites of endothermic hosts should not be strongly influenced by the outside thermal environment.

Biotic interactions not involving the host itself can also drive the evolution of parasite body sizes. Firstly, parasites may attain higher intensities of infection (i.e. higher numbers of conspecific parasites per individual host) in the tropics than in temperate areas, leading to greater intraspecific competition for host resources. Parasite species occurring at higher average intensities may generally have smaller body sizes; this is true for at least some ecto- [41] and endo-parasites [42]. This is not just phenotypic plasticity in response to immediate competition, but instead appears to be a fixed, adaptive adjustment in body size in the face of sustained competition. There is evidence of a latitudinal cline in infection intensities in some host–parasite systems [43], with higher intensities observed at low latitudes, but not in others [44]. Thus, latitude-dependent parasite crowding may only contribute to a Bergmann-type pattern in some taxa.

Secondly, parasites have their own predators, whose prey detection and selection may be size-dependent [45]. In the case of external parasites, natural selection may favour parasites smaller than those that could be sustained by available host energy in order to avoid death by host preening (e.g. [46]). Other types of predation on parasites might contribute to latitudinal gradients in parasite body size if they vary with latitude. For instance, the frequency and intensity of cleaning interactions, during which specialized cleaner organisms remove ectoparasites from fish, appear to be greater in the tropics than at higher latitudes [47,48]; the same is true for birds that specialize on feeding on ticks taken from their mammalian hosts [49]. Cleaning-mediated selection for predation avoidance and small body sizes could reinforce the action of other factors and help give rise to the pattern predicted by Bergmann's rule.

Any or all of the hypotheses in table 1 can *a priori* generate a Bergmann's rule-type latitudinal pattern. What is the empirical evidence for such a pattern among parasites? A search of the Web of Science database was conducted to find comparative studies that have tested for a latitude versus body size relationship among parasite species (see search details and inclusion criteria in electronic supplementary material). Only 15 comparative analyses, from 11 separate studies, were found that met all inclusion criteria (table 2). Since there are few of them, and because they use vastly different analytical approaches and measures of body size, and also report different types of effect sizes, only their qualitative results are considered; conducting a formal quantitative meta-analysis was not an option. When the authors of a study analysed their data in two different ways, i.e. with and without accounting for the effect of an important confounding variable like parasite phylogeny or host body size, the test accounting for confounding variables was chosen as a more conservative option. Very few of the available studies controlled for the potential effect of host body size, making it difficult to distinguish a mere host size­–parasite size correlation from other processes directly associated with Bergmann's rule. Having said that, almost half (7 of 15) of the analyses reported a positive latitude–body size relationship, thus supporting Bergmann's rule, whereas only one went against the rule with a negative relationship. There is no clear discrepancy in the type of relationship observed between studies on ecto- versus endoparasites, or vertebrate versus invertebrate hosts. Any conclusion must be tempered with caution, however; publication bias may have resulted in negative or inconclusive relationships being less likely to be published than positive ones (the ‘file drawer’ effect), a limitation that also applies to the findings summarized in the following sections.

**Table 2.** Empirical studies (comparative analyses) evaluating latitudinal gradients in functional traits of parasites.

parasite taxon	host taxon	latitudinal extent	trait measure	confounding variables considered	relationship with latitude	sample size^a^	reference
*body size* (*Bergmann's rule*)							
helminths	vertebrates	0°–76° (across whole world)	body length	—	negative	202	[50]
trematodes	vertebrates	0°–70° (across whole world)	body surface area	phylogeny	non-significant	1008	[51]
trematodes	ectothermic vertebrates	0°–70° (across whole world)	body surface area	phylogeny	non-significant	632	[51]
trematodes (cercarial stage)	gastropods	2°–63° (across aquatic habitats)	body volume	taxonomy	non-significant	340	[52]
				host body size			
monogeneans	fish and amphibians	3°–50° (across whole world)	body length	phylogeny	positive	613	[53]
cestodes	elasmobranchs (sharks)	1°–73° (across all oceans)	body length	host body size	positive	80	[54]
cestodes	elasmobranchs (batoids)	1°–73° (across all oceans)	body length	host body size	non-significant	102	[54]
copepods	fish and invertebrates	0°–72° (across aquatic habitats)	body length	phylogeny	positive	696	[55]
isopods	fish and invertebrates	13°–42° (across all oceans)	body length	phylogeny	positive	224	[56]
amphipods	marine mammals and invertebrates	9°–62° (across aquatic habitats)	body length	phylogeny	positive	218	[57]
ascothoracidans	invertebrates	0°–57° (across all oceans)	body length	phylogeny	positive	59	[58]
rhizocephalans	crustaceans	2°–75° (across all oceans)	body length	phylogeny	non-significant	91	[58]
mesostigmatid mites	animals	0°–75° (across whole world)	body length	—	positive	1254	[59]
all parasitoid wasps	insects	0°–66° (across whole world)	body length	phylogeny	non-significant	474	[60]
ichneumonid parasitoid wasps only	insects	0°–66° (across whole world)	body length	phylogeny	non-significant	255	[60]
*host specificity* (*MacArthur's rule*)							
trematodes	fish	4°–70° (across all oceans)	no. host species	—	positive	1365	[61]
monogeneans	fish	4°–70° (across all oceans)	no. host species	—	non-significant	624	[61]
fleas	small mammals	34°–61° (across the Palaearctic)	no. host species	phylogeny	non-significant	120	[62]
fleas	small mammals	34°–61° (across the Palaearctic)	host taxonomic distinctness	phylogeny	positive	120	[62]
*geographic range* (*Rapoport's rule*)							
helminths	humans	5°–65° (across North Hemisphere)	latitudinal extent	excluding rare species	positive	41	[63]
helminths	humans	5°–40° (across South Hemisphere)	latitudinal extent	excluding rare species	positive	18	[63]
trematodes	fish	33°–65° (across Europe)	range area (km^2^)	phylogeny	hump-shaped	67	[64]
				continental shape			
trematodes	amphibians	33°–65° (across Europe)	range area (km^2^)	phylogeny	hump-shaped	50	[64]
				continental shape			
trematodes	reptiles	33°–65° (across Europe)	range area (km^2^)	phylogeny	positive	18	[64]
				continental shape			
trematodes	mammals	33°–65° (across Europe)	range area (km^2^)	phylogeny	hump-shaped	93	[64]
				continental shape			
trematodes	birds and mammals	33°–65° (across Europe)	range area (km^2^)	phylogeny	hump-shaped	29	[64]
				continental shape			
trematodes	birds	33°–65° (across Europe)	range area (km^2^)	phylogeny	hump-shaped	307	[64]
				continental shape			
fleas	small mammals	34°–61° (across the Palaearctic)	range area (km^2^)	phylogeny	positive	120	[61]
*latitudinal gradient in egg size*							
helminths	vertebrates	0°–76° (across whole world)	egg length	—	negative	202	[50]
trematodes	vertebrates	0°–70° (across whole world)	egg length × width	phylogeny	non-significant	1008	[51]
				parasite body size			
trematodes	ectothermic vertebrates	0°–70° (across whole world)	egg length × width	phylogeny	non-significant	632	[51]
				parasite body size			
trematodes (cercarial stage)	vertebrates	2°–63° (across aquatic habitats)	egg volume	taxonomy	non-significant	237	[52]
				host body size			
copepods	fish and invertebrates	0°–72° (across aquatic habitats)	egg diameter	phylogeny	negative	696	[55]
				parasite body size			
ascothoracidans	invertebrates	0°–57° (across all oceans)	egg diameter	phylogeny	non-significant	59	[58]
				parasite body size			
rhizocephalans	crustaceans	2°–75° (across all oceans)	egg diameter	phylogeny	non-significant	91	[58]
				parasite body size			

^a^Number of taxa (though not necessarily the same as the number of independent observations) included in the comparative analysis.

The weak and inconsistent tendency for comparative studies in table 2 to support Bergmann's rule is mirrored by the three relevant studies that did not meet inclusion criteria. First, a study of camallanid nematodes found that species in temperate areas were larger than tropical congeneric species [65]. Second, a comparative study on ticks found no correlation between body size and local annual mean temperature, which itself varies with latitude [66]. Finally, a comparison across communities of fleas infecting small mammals in Mongolia reported that the average parasite body size, computed across all locally occurring species, increased toward higher latitudes and lower temperatures [67].

## 3. Host specificity and MacArthur's rule

The arguments first proposed by MacArthur [25] and later elaborated by Vázquez & Stevens [24] to explain the latitudinal gradient in niche breadth provide a mechanism applicable to parasites. In brief, lower environmental variability and higher productivity in the tropics should allow populations to remain more stable and temporally predictable. In principle, if a host population remains predictably available over time, parasites can specialize on fewer host species to avoid the need to evolve costly adaptations (e.g. immune evasion mechanisms) against multiple host species. Although the assumptions that environmental conditions and population abundances are more stable in the tropics are questionable, as is the universality of the latitudinal gradient in niche breadth [24,68], the hypothesis remains plausible for parasites (table 1). The other mechanistic explanation for reduced niche breadth in the tropics invokes interspecific competition and ‘species packing’ acting to reduce niche overlap because of the greater diversity of species at low latitudes [25]. Since the number of parasite species per host species does not increase toward lower latitudes for all types of host–parasite associations [9], this cannot be the universal explanation, though it may play a role in some cases.

Two confounding variables may obscure the latitudinal gradient in host specificity, if it exists. Firstly, the host specificity of parasites shows a significant phylogenetic signal, i.e. it is conserved and therefore closely related species tend to exploit a similar number of host species, or a similar phylogenetic diversity of host species [69]. The taxonomic composition of parasite assemblages differs among latitudes, and so will their inherited degree of host specialization. Secondly, the range of host species used by a parasite is dependent to some extent on the pool of locally available host species [70,71]. The generally higher diversity of free-living taxa in the tropics may therefore counteract any tendency for greater specialization at low latitudes, by offering tropical parasites more host options.

With these caveats in mind, a search of the Web of Science database for comparative studies testing for a latitudinal gradient in host specificity among parasite species (see electronic supplementary material) yielded only four analyses, from two separate studies (table 2). Two of these analyses report a positive relationship between latitude and either the number of host species used or their taxonomic distinctness, thus supporting MacArthur's rule, while the other two report no significant pattern. Not included in table 2 are a few studies which did not measure host specificity directly for individual parasite species, but instead calculated measures of niche breadth from entire host–parasite interaction networks and related those with latitude [72–74]. These studies also found either no or inconsistent support for a latitudinal gradient in host specificity. Overall, the few available studies provide limited evidence in support of MacArthur's rule, but report no trends running completely counter to its prediction.

All else being equal, with the spatial ranges of different potential host species overlapping partially but not completely, if a parasite is a generalist exploiting many host species, its geographical range should inevitably be larger than that of a specialist parasite exploiting only one of those host species. Indeed, positive interspecific correlations are often found between the geographical range sizes of parasites and the number of host species they exploit (the inverse of host specificity) [75,76], suggesting that MacArthur's rule and Rapoport's rule (which is discussed next) are not fully independent of each other, at least when applied to parasites.

## 4. Parasite geographical range and Rapoport's rule

Among free-living organisms, the positive correlation between latitude and the size of the geographical range proposed by Rapoport [27] appears consistently among studies performed in the Northern Hemisphere, but less so for studies from the Southern Hemisphere or studies conducted on global scales [77]. As explained above, the most parsimonious explanation for the existence of a latitudinal gradient in geographical range size in parasites would be that parasite range sizes mirror those of their main host, or the superimposed ranges of all their hosts [78,79]. If host organisms follow Rapoport's rule, then so would their parasites (table 1). The link between the distribution of an essential resource and that of its consumer is therefore the simplest mechanism to explain parasite geographical ranges [13].

Among the other hypotheses proposed to account for Rapoport's rule [29], only one seems appropriate for parasites. The climate variability hypothesis [28,80] states that species occurring in abiotically variable habitats, such as those at high latitudes, have been selected to have wider environmental tolerances, allowing them to expand their geographical ranges further than species from relatively stable habitats like the tropics. Assuming they are not restricted by very narrow host specificity, this could apply to all parasites, even endoparasites of endothermic hosts since they have external transmission stages exposed to environmental conditions. Other hypotheses, from the existence of hard boundaries between biogeographic biomes [29] to possible latitudinal gradients in dispersal abilities [81], are either related to the connection between host and parasite range sizes mentioned earlier, or implausible for parasites.

As with host specificity and many other parasite traits, the geographical range sizes of parasites may be phylogenetically conserved, as parasite species within the same clade tend to share similar host taxa and similar environmental tolerances [82]. Therefore, not only should comparative analyses of geographical range sizes control for phylogenetic influences, but also the uneven spatial distribution of species from different genera, families or orders across the globe which can mask any pattern fitting Rapoport's rule.

A search of the Web of Science database for comparative studies testing for a latitudinal gradient in geographical range sizes among metazoan parasite species (see electronic supplementary material) yielded only nine analyses, from three separate studies (table 2). At first glance, these studies offer some support for Rapoport's rule. Those that do not report a positive association between latitude and geographical range size report instead a hump-shaped relationship. However, these analyses were conducted in Europe, and the shape of the European continent constrains the shape and sizes of geographical ranges [64]; therefore, the humped pattern does not necessarily contradict Rapoport's rule. Of note is a study of human parasites, which reports increases in the geographical range sizes of helminths as a function of latitude [63]. The same study found the same pattern for bacterial, fungal and protozoan parasites. As all of these share the same host species that has a global distribution, the climate variability hypothesis seems to provide a mechanism capable of explaining the observed pattern: parasite species evolving at higher latitudes and selected to tolerate (and transmit under) a broader range of conditions achieve greater dispersal. However, latitudinal differences in human social, cultural, and economic factors could also account in part for this apparent case of Rapoport's rule. For instance, while generally better access to medical assistance and greater investments in sanitation and water quality in temperate countries might tend to counteract parasite spread, more frequent international travel between those countries might contribute to maintaining broad parasite geographical ranges. In any event, the available studies provide some support for Rapoport's rule applying to parasites, and no evidence against it.

## 5. Latitudinal gradients in other parasite traits

Functional traits have recently been promoted as a source of new insights into parasite diversity and community ecology [83]. Trait-based metrics can indeed capture different aspects of parasite assemblages than species-based ones. However, many of the traits thought to be informative for parasite communities consisting of representatives from many parasite phyla [83] show little variation within phyla; they are therefore not useful in within-clade comparative analyses testing for latitudinal gradients in functional traits. For example, the type of life cycle or the site of attachment is usually the same among parasite species belonging to the same class, or even phylum.

One of the proposed ‘standard’ functional traits of parasites [83], egg size, has received attention in the context of a latitudinal gradient. There is some evidence for a latitudinal gradient in egg sizes among free-living ectotherms, but the pattern is far from universal (e.g. [84–87]). For marine benthic invertebrates, the tendency for egg sizes to increase toward high latitudes has been called Thorson's rule [88]. For parasites, the abundance of resources available by feeding on a much larger host may relax the trade-off between egg number and egg size [89]. However, parasites are unlikely to fully escape from this reproductive compromise [30]. Comparative studies have also demonstrated that in general, larger-bodied parasite species produce larger eggs, though there are exceptions [51,55,58]. Therefore, whenever a clade of parasites follows Bergmann's rule, we might expect it to also display a latitudinal gradient in *absolute* egg sizes. This is the simplest hypothesis regarding a latitudinal gradient in egg sizes (table 1). Of greater interest would be a latitudinal gradient in *relative* egg size, i.e. controlling for body size, thus revealing whether parasites invest proportionally more into each egg at higher than at lower latitudes. This is predicted by the hypothesis that animals produce larger offspring at high latitudes as a strategy to reduce development time and mortality risk at colder temperatures [90].

A search of the Web of Science database (see electronic supplementary material) found seven comparative analyses, from five separate studies, testing for a latitude versus egg size relationship among parasite species (table 2). Most control for the potential influence of parasite body size on egg sizes, and overall they provide no support for an increase in egg sizes toward high latitudes. Although these analyses cannot rule out that *absolute* egg sizes are larger at higher latitudes if parasite body sizes are also larger, they do rule out latitudinal trends in *relative* egg sizes.

A study of variability in egg sizes (i.e. departures from the mean egg size) among trematode species revealed a clear negative relationship with latitude, mostly driven by species that release their eggs in terrestrial environments [91]. In other words, within-species egg sizes are much more homogeneous in colder temperate habitats than in the tropics. This suggests that trematodes may allocate resources more evenly among their eggs in more challenging conditions, rather than producing larger eggs [91].

## 6. Future directions

The present review has highlighted the scarcity of studies to date that have explored geographical patterns in the distribution of key parasite functional traits. To remedy our limited knowledge, we need not only more studies, but better studies. Here, I propose three steps toward more robust testing of ecogeographical rules in parasites.

First, there is a need for additional datasets covering a broader taxonomic range of both hosts and parasites, and assembled for the specific purpose of testing geographical patterns in trait distribution. There exist excellent host–parasite databases, such as the Global Mammal Parasite Database [92]. However, these have been compiled for different purposes; they may have inherent biases making them unsuitable to study parasite trait biogeography, therefore using them for this purpose may lead to artefactual patterns. The other risk of relying on the few existing databases is that their repeated use does not produce independent tests; indeed, several entries in table 2 actually use overlapping datasets.

Second, analytical approaches used to test for ecogeographical rules in parasites need greater sophistication. This review has uncovered a predominance of host-mediated effects among published studies. However, no study to date has successfully disentangled the influence of host properties from the direct influence of latitude or its associated bioclimatic factors on the global distribution of parasite traits. The null expectation would be that once the effects of host properties are negated, there is no independent influence of latitude on parasite traits. There exist promising analytical frameworks that can be applied to large-scale databases of parasite traits when accompanying data are also available on host traits, host and parasite phylogenies, and latitude or environmental variables. For example, if parasite traits are indeed, to some extent, the product of host traits while at the same time being constrained by the parasites' own evolutionary history, there are comparative methods that allow for the simultaneous inclusion of host and parasite phylogenies as separate causative factors of any focal trait [93]. This approach would allow one to determine to what extent host traits matter in shaping the geography of parasite traits. Alternatively, and perhaps more promising, structural equation modelling provides a powerful way to quantify direct and indirect causal pathways among multiple variables, simultaneously testing multiple causal hypotheses while incorporating phylogenetic information and random effects [94]. This approach would be perfect to tease apart the respective influence of latitude itself versus that of host traits (i.e. host body size in the case of Bergmann's rule, local host species richness in the case of MacArthur's rule) on parasite traits.

Finally, the existing evidence is purely correlational in nature; it would be greatly strengthened with the use of experimental approaches. For instance, multi-generational (serial passage) experiments, with parasites allowed to evolve under a range of thermal regimes or other conditions linked to latitude, would allow stronger causal inference. Although logistically challenging and unlikely to include multiple parasite species, such experiments would at least provide a solid test of the mechanistic processes presumed to link latitude with key parasite properties.

## 7. Synthesis and conclusion

Parasite species richness per host species does not consistently peak at low latitudes across all parasite types [9]; the same can be said of their abundance (number of parasite individuals per host individual) [43,44] and their negative impacts on host fitness [95,96]. The present review confirms that parasites also do not consistently follow Bergmann's rule, MacArthur's rule, or Rapoport's rule about latitudinal gradients in body size, host specificity and geographical range sizes, respectively. The number of available studies and the range of parasite taxonomic groups investigated to date remain limited. Still, based on the evidence presently available, there is no clear distinguishing feature associated with studies that support the rules versus those that do not with respect to the host or parasite taxa involved, mode of transmission, site of parasite attachment, etc.

In the cases where parasites were found to follow the ecogeographical rules, the most parsimonious explanation may be simply that their ecological traits track those of their hosts. In other words, most geographical patterns in parasite traits are probably epiphenomena of host biogeographic patterns. The chain of causality appears to go from hosts to parasites. If species within a higher host taxon follow Bergmann's rule and achieve larger sizes at higher latitudes, their larger sizes will drive the evolution of larger parasites, which produce larger eggs (in absolute if not relative terms). If host species follow Rapoport's rule and occupy larger geographical ranges at higher latitudes, for any given level of host specificity their parasites will also automatically have larger geographical ranges. Parasite traits are not always just a consequence of host traits, of course; they can also be shaped by bioclimatic factors related to latitude. For instance, the greater host specificity (narrower niche breadth) of tropical parasites may be a direct response to more stable host populations at low latitudes due to limited climatic variability. Similarly, the tolerance of parasite eggs or dispersal stages to environmental conditions can play a role in determining the geographical distribution of parasite species. Nevertheless, when parasitic organisms follow classical ecogeographical rules (often they do not), the overarching process appears to be for parasite functional biogeography being driven by host functional biogeography, as a reflection of the intimate interaction between parasites and their hosts, and the unidirectional dependence of the former on the latter.

In the absence of the kind of robust tests proposed in the preceding section, and although the evidence that parasites follow Bergmann's rule, MacArthur's rule and Rapoport's rule is weak at best, it seems reasonable to postulate that any latitudinal gradient of parasite traits may simply follow the underlying latitudinal cline in their host resources. The tight coevolutionary history of parasites and their hosts has shaped patterns of codiversification and coadaptation [97]. It may also have shaped the geography of parasite functional traits, with the hosts providing a template. In the absence of a rigorous test of this idea, it remains the most parsimonious universal explanation for the (far from universal) latitudinal gradients in parasite traits.

## Data accessibility

This article has no additional data.
